# Without Salt, the ‘Thermophilic’ Protein Mth10b Is Just Mesophilic

**DOI:** 10.1371/journal.pone.0053125

**Published:** 2012-12-27

**Authors:** Nan Zhang, Xian-Ming Pan, Meng Ge

**Affiliations:** 1 Ministry of Education Key Laboratory of Bioinformatics, School of Life Sciences, Tsinghua University, Beijing, People's Republic of China; 2 CAS Key Laboratory of Genome Sciences and Information, Beijing Institute of Genomics, Chinese Academy of Sciences, Beijing, People's Republic of China; Aligarh Muslim University, India

## Abstract

Most proteins from thermophiles or hyperthermophiles are intrinsically thermostable. However, though *Methanobacterium thermoautotrophicum ΔH* is a thermophilic archaeon with an optimal growth temperature of 65°C, Mth10b, an atypical member the Sac10b protein family from *M. thermoautotrophicum ΔH*, seems not intrinsically thermostable. In this work, to clarify the molecular mechanism of Mth10b remaining stable under its physiological conditions, the thermodynamic properties of Mth10b were studied through equilibrium unfolding experiments performed at pH 7.0 monitored by circular dichroism (CD) spectra in detail. Our work demonstrated that Mth10b is not intrinsically thermostable and that due to the masking effect upon the large numbers of destabilizing electrostatic repulsions resulting from the extremely uneven distribution of charged residues over the surface of Mth10b, salt can contribute to the thermostability of Mth10b greatly. Considering that the intracellular salt concentration is high to 0.7 M, we concluded that salt is the key extrinsic factor to Mth10b remaining stable under its physiological conditions. In other word, without salt, ‘thermophilic’ protein Mth10b is just a mesophilic one.

## Introduction

Organisms permeate almost everywhere on earth, including in harsh environments of extreme temperature, pH, salinity and pressure. Extremophiles are defined as organisms that flourish in habitats of extreme conditions. Among extremophiles, organisms adapted to high temperatures are called thermophiles, which grow optimally between 50 and 80°C, and hyperthermophiles, which grow optimally above 80°C.

Proteins from thermophiles and hyperthermophiles are usually exhibit intrinsically remarkable thermostability and resistance to chemical denaturants [1]–[3]. Considerable efforts have been invested during the past several decades to understand how thermophilic and hyperthermophilic proteins can maintain stable at high temperatures. Enough experimental evidence has been accumulated to conclude that no single mechanism is responsible for the remarkable stability of thermophilic and hyperthermophilic proteins [1]–[3]. Thermostability of these proteins appears to be implemented by a variety of strategies such as increased number of salt bridges, improved core packing, greater rigidity, increased number of prolines, additional hydrogen bonds, and higher states of oligomerization [4]–[21]. Moreover, though most thermophilic and hyperthermophilic proteins are intrinsically thermostable, apart from the above mentioned intrinsic factors, some extrinsic factors, such as macromolecular crowding, coenzymes, substrates, some sugar-derivatives, and salts, also have been demonstrated to contribute to protein thermostability in the context of a biological cell [22]–[27].

The Sac10b protein family is generally regarded as a group of DNA-binding proteins that is highly conserved and widely distributed within the archaea. Typical members of this family are small basic homodimeric proteins which bind to DNA without sequence specificity [28]–[35]. Recently, Mth10b, an atypical member of the Sac10b protein family was identified from *M. thermoautotrophicum ΔH* by our laboratory [36], [37]. Though it is similar to typical Sac10b family proteins with respect to its primary, secondary, tertiary structure and in its preferred oligomeric forms, unlike typical Sac10b family proteins, Mth10b is an acidic one with potential isoelectric point of 4.56 and bind to neither DNA nor RNA *in vitro* [36], [37]. When we try to purify recombinant Mth10b from *Escherichia coli* through maintaining cells lysate at high temperature, an interesting phenomenon was found: though *M. thermoautotrophicum ΔH* is a typical thermophilic archaeon that grows at temperatures in the range of 40–70°C, with an optimal temperature of 65°C [38], the recombinant Mth10b was precipitated absolutely with those unwanted proteins after the cells lysate was maintained at 60°C for 20 minutes [36]. This phenomenon suggests that Mth10b is not an intrinsically thermophlic protein and that there should be some extrinsic factors can help Mth10b remaining stable *in vivo*.

In order to clarify why Mth10b can maintain stable under its physiological conditions, in the present work, we studied the thermodynamic properties of Mth10b through denaturant-induced unfolding and heat-induced unfolding monitored by circular dichroism (CD) spectra in detail. Our results demonstrated that Mth10b is not intrinsically thermostable and that salt is the key factor to Mth10b remaining stable under the physiological conditions.

## Materials and Methods

### Materials

HEPES, GdnHCl, Urea, Tris, and isopropyl β-D-thiogalactoside (IPTG) were purchased from Sigma. The expression plasmid pET11a-*mth10b* containing the *mth10b* gene, and host strains *E. coli* DH5α and BL21 (DE3) were from our laboratory stocks. All chromatography apparatus and materials were purchased from GE Healthcare.

### Protein preparation

The Mth10b protein was expressed in *E. coli* and purified to homogeneity as previously described [36]. Protein purity was higher than 95% as confirmed by 15% SDS-PAGE. The purified protein samples were dialyzed against 50 mM NH_4_HCO_3_ then lyophilized and stored at −20°C.

### Unfolding studies

Unfolding of Mth10b was studied by taking CD measurements, performed in basic running buffer (10 mM HEPES/pH 7.0) containing different concentrations of NaCl, with a Pistar-180 spectrometer with a Peltier temperature-controlled cell holder. The CD signal was monitored using a rectangular quartz cuvette with a path length of 1 mm.

For denaturant-induced unfolding, two most common chemical denaturants, GdnHCl and urea were employed respectively. The samples containing various concentrations of denaturant (GdnHCl or urea) were equilibrated at 25°C overnight and then measured by far-UV CD at 222 nm with an averaging time of 1 min. Reversibility of denaturant-induced unfolding was checked by diluting denatured protein in a high denaturant concentration into the buffer solution and comparing the CD spectrum with that of the native protein. CD spectra were scanned at 1 nm intervals from 200 (or 205) to 250 nm. Moreover, the urea stock solution was freshly prepared on the day of use.

For heat-induced unfolding, measurements were carried out in the presence of different concentrations of GdnHCl. Each sample was heated with a stepwise change of 2°C, and the far-UV CD signal at 222 nm was recorded, with a 2 min equilibration time, and a 1 min averaging time at each temperature point. Reversibility was checked by returning to the beginning temperature and comparing the CD spectrum with the premelt spectrum. CD spectra were scanned at 1 nm intervals from 205 to 250 nm.

### Analysis of the denaturation data

Thermodynamic properties of Mth10b were calculated assuming a two-state denaturation process: 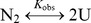. The observed equilibrium constant (*K*_obs_) and the corresponding free energy change (Δ*G*) of unfolding at temperature *T* or denaturant concentration [*D*] were calculated according to:
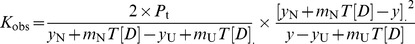
(1)

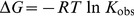
(2)where *P*_t_ is the total protein concentration in monomer units; *R* is the gas constant; *T* is the absolute temperature; *y* is the experimentally measured signal value at a given temperature (*T*) or given denaturant concentration ([*D*]); 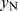 and 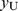 are the intercepts; and 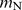 and 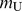 are the slopes of the native and unfolded baselines respectively.

According to the linear free energy model [39]–[41], free energy changes (Δ*G*), enthalpy changes (Δ*H*) and entropy changes (Δ*S*) during protein unfolding are expected to vary linearly with denaturant concentration ([*D*]):
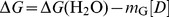
(3)

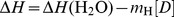
(4)

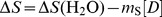
(5)where Δ*G*(H_2_O), Δ*H*(H_2_O) and Δ*S*(H_2_O) represent the free energy, enthalpy and entropy changes of unfolding in the absence of denaturant; 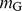, 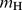 and 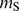 are the slopes of the transition for the free energy enthalpy and entropy changes respectively. The denaturant concentration at the transition midpoint (*C*_m_), where 50% of the protein is unfolded, is a function of the protein concentration and can be calculated according to:
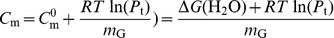
(6)Where 
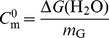
 is the denaturant concentration at the transition midpoint at protein monomer concentration of 1 M (*P*_t_ = 1 M).

For thermal unfolding, the free energy change (Δ*G*_T_) of unfolding at temperature (*T*) can be represented as:
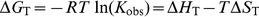
(7)where Δ*H*_T_ and Δ*S*_T_ are the enthalpy and entropy changes of unfolding at temperature (*T*), respectively. Assuming that the heat capacity change (Δ*C*_p_) between the native and unfolded states of the system is relatively independent of temperature, the temperature dependences of Δ*H*_T_ and Δ*S*_T_ can be calculated according to:
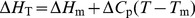
(8)

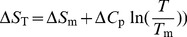
(9)where Δ*H*_m_ and Δ*S*_m_ are the enthalpy and entropy changes of the protein at the transition midpoint, where *T* = *T*_m_. Substituting Equations 8 and 9 into Equation 7 gives the following named Gibbs-Helmholtz equation [41], [42]:
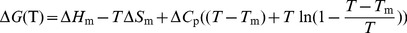
(10)Within the transition range, where 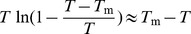, therefore Equation 7 can be simplified to the van't Hoff plot:
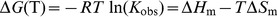
(11)The temperature of the transition midpoint (*T*_m_), also named melting temperature, is a function of protein and denaturant concentration and can be calculated according to:
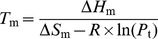
(12)Substituting Equations 4 and 5 into Equation 12 gives:
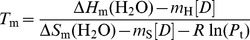
(13)


## Results

### CD spectra characterization of Mth10b and unfolding reversibility

**Figure 1:**
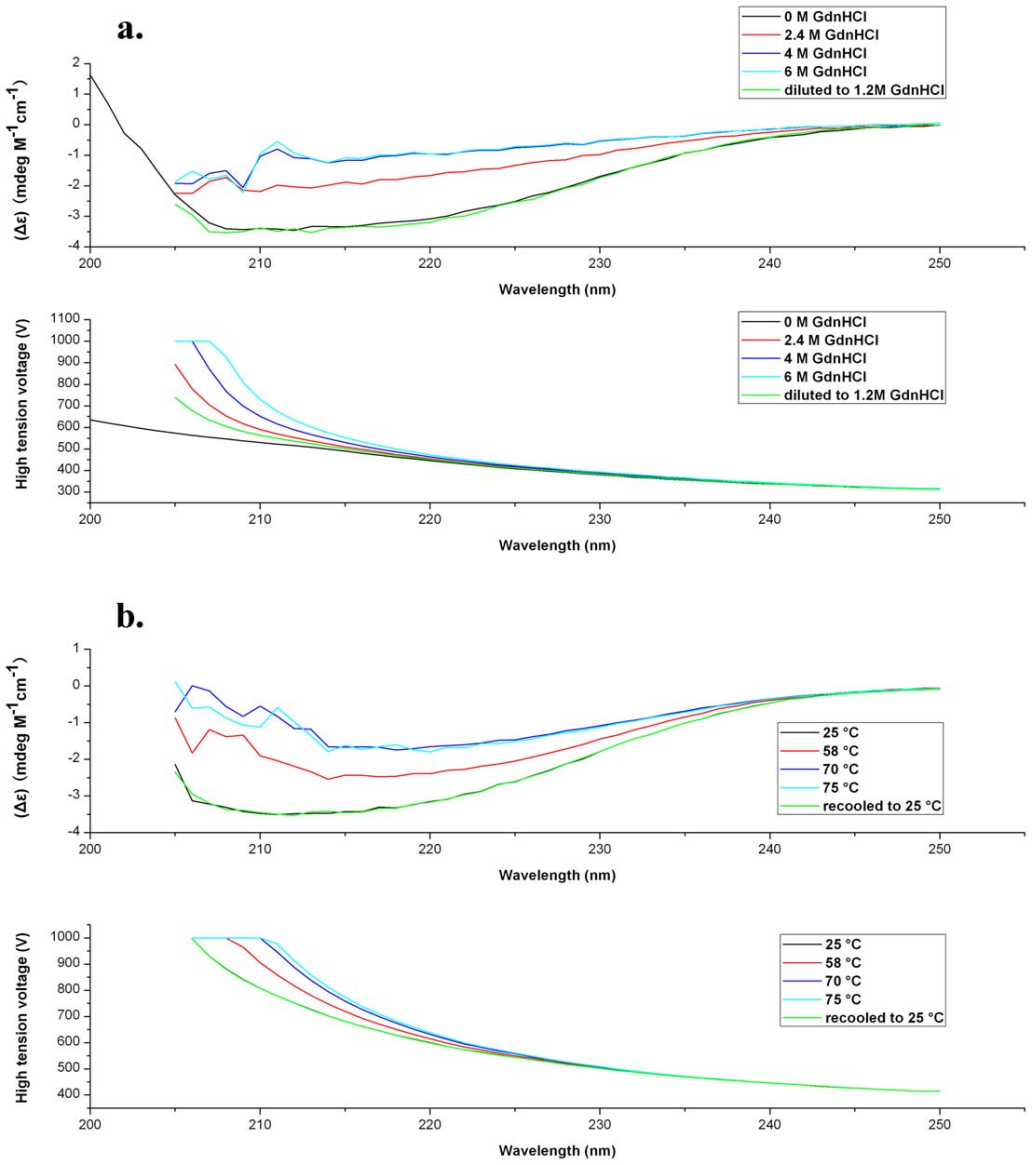
CD spectra characterization of Mth10b and unfolding reversibility. a. GdnHCl-induced unfolding in basic running buffer performed with 15 µM Mth10b at 25°C: the black trace is the spectrum of the native protein recorded in the absence of GdnHCl; the red, blue and cyan traces are the spectra recorded in the presence of 2.4, 4, and 6 M GdnHCl; the green trace is the spectrum for 5-fold dilution of 6 M GdnHCl denatured protein. b. Heat-induced unfolding in basic running buffer performed with 30 µM Mth10b in the presence of 1.2 M GdnHCl recorded at 25°C (the black trace), 60°C (the red trace), 70°C (the blue trace) and 75°C (the cyan trace).The green trace is the spectrum of Mth10b after heat-induced unfolding at 75°C, followed by re-equilibration at 25°C, which is almost identical to that of premelting Mth10b (the black trace).

As far-UV CD spectrum of a protein conforms to its secondary structure, in this work, CD was employed to monitor unfolding of Mth10b. Far-UV CD spectra of Mth10b recorded in basic buffer containing different concentrations of GdnHCl are shown as traces in different colors in Figure 1a. The black trace shows typical CD spectrum of native Mth10b recorded at 25°C, agreeing well with that reported previously [36]. As shown in the figure, the CD signal of Mth10b reduced to about 50% in the presence of 2.4 M GdnHCl (the red trace); Mth10b lost about 70% CD signal in the presence of 4.0 M GdnHCl (the blue trace). The CD spectrum of denatured Mth10b was found no further change when the concentration of GdnHCl was increased to 6.0 M (the cyan trace). Residual CD signal suggested the existence of a compact denatured state of Mth10b. Moreover, the green trace represents a typical CD spectrum for 5-fold dilution of 6.0 M GdnHCl denatured protein, which is almost identical to that of native Mth10b, indicating that GdnHCl-induced unfolding of Mth10b is fully reversible. Similarly, urea-induced unfolding of Mth10b is also fully reversible and there also exist a similar compact denatured state of Mth10b (data not shown).

Due to Mth10b aggregated irreversibly at temperatures higher than 50°C, which is agreeing well with our previous report [36], heat-induced unfolding of Mth10b in basic running buffer could not be performed. However, we found that when the sample contains higher than 0.8 M GdnHCl, fully reversible thermal unfolding of Mth10b can be achieved successfully. Therefore, in this work, the thermal unfolding of Mth10b was carried out in the presence of different concentrations of GdnHCl. Figure 1b shows typical CD spectra of Mth10b in basic running buffer containing 1.2 M GdnHCl recorded at different temperatures. Compared with the spectrum recorded at 25°C (the black trace), when the temperature was increased to about 60°C, Mth10b lost about a quarter of CD signal (the red trace); when the temperature was increased to about 70°C, Mth10b lost about a half of CD signal (the blue trace); when the temperature was further increased to about 75°C, the spectrum displayed no obvious further change (the cyan trace). More residual signal than that existed in results of GdnHCl-induced unfolding performed at room temperature indicated that there is exist a different compact denatured state of Mth10b. This may be explained by that the two unfolding pathways are entirely different. The green trace in Figure 1b shows a typical CD spectrum after heat-induced unfolding at 75°C in the presence of 1.2 M GdnHCl, followed by re-equilibration at 25°C, which is almost identical to that of premelting Mth10b (the black trace).

### Denaturant-induced unfolding

**Figure 2:**
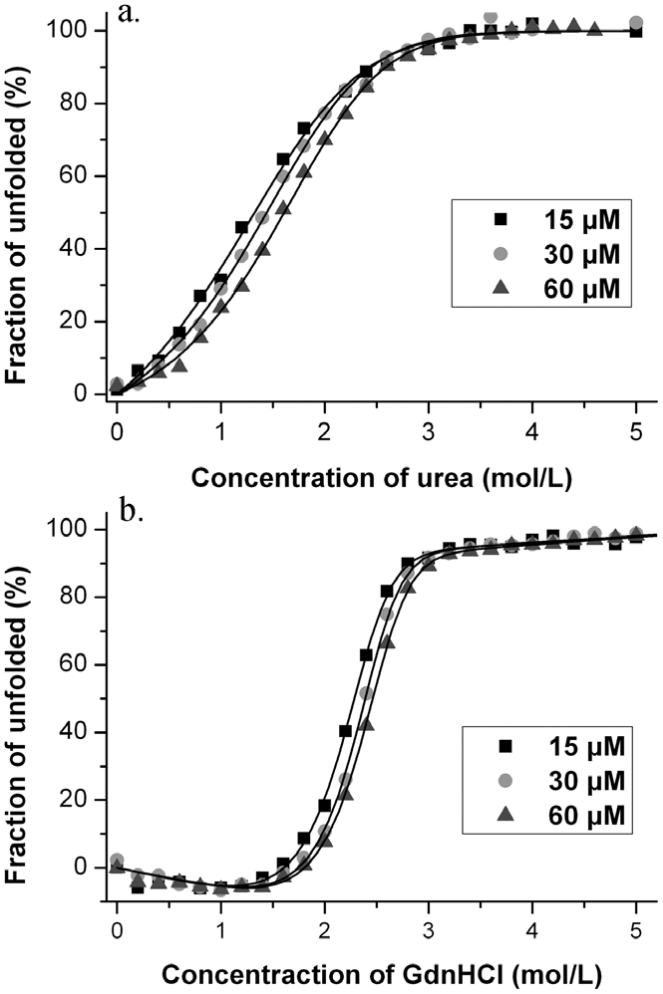
Denaturant-induced unfolding profiles of Mth10b in basic running buffer performed at 25°C. a. Urea-induced unfolding; b. GdnHCl-induced unfolding. The continuous lines represent the fit of the experimental data to the model described in methods.

Denaturant induced unfolding of Mth10b was studied in basic running buffer at 25°C by far-UV CD monitored at 222 nm. In this work, urea and GdnHCl were chosen as the denaturant for unfolding, respectively. Figure 2a shows representative urea-induced unfolding profiles of Mth10b performed at different protein concentrations in basic running buffer (10 mM HEPES/pH 7.0). The unfolding transitions appeared to be monophasic, suggesting the absence of a folding intermediate in the urea-induced unfolding process of Mth10b. Moreover, good protein concentration dependence of the transition curves was observed. Assuming a two-state transition and a linear dependence of Δ*G* on urea concentration, the urea-induced unfolding profiles obtained from measurements at three different concentrations (15 µM, 30 µM and 60 µM) were analyzed. The resulting Δ*G*(H_2_O) at 25°C were 34.5±0.8 kJ·mol^−1^, 34.5±0.7 kJ·mol^−1^ and 34.7±0.5 kJ·mol^−1^, respectively; the resulting C_m_ at 25°C were 1.08±0.07 mol·L^−1^, 1.30±0.04 mol·L^−1^ and 1.51±0.03 mol·L^−1^, respectively.

Figure 2b shows representative GdnHCl-induced unfolding profiles of Mth10b performed at different protein concentrations in basic running buffer (10 mM HEPES/pH 7.0). Interestingly, the resistance of Mth10b to GdnHCl is much stronger than to urea. Similar with urea-induced unfolding profiles, the transition curves were characterized by a single sharp change in the ellipticity without any detected intermediates which seems like a typical two-state transition. The transition curves of GdnHCl-induced unfolding also displayed good dependence on protein concentration. The linear free energy model was used to analyze the GdnHCl-induced unfolding profiles. Measurements performed at protein concentrations of 15 µM, 30 µM and 60 µM gave the Δ*G*(H_2_O) values of 63.0±2.9 kJ·mol^−1^, 64.0±2.7 kJ·mol^−1^ and 62.9±1.8 kJ·mol^−1^, and the C_m_ values of 2.19±0.01 mol·L^−1^, 2.30±0.01 mol·L^−1^ and 2.36±0.01 mol·L^−1^, at 25°C.

### Heat-induced unfolding

**Figure 3:**
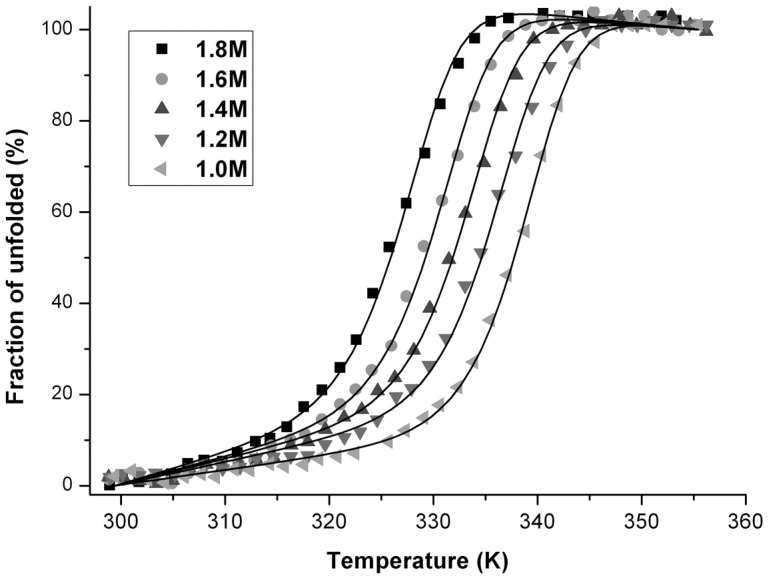
Heat-induced unfolding profiles of 30 µM Mth10b performed in the presence of GdnHCl: 1.0 M (▪), 1.2 M (•), 1.4 M (▴), 1.6 M (▾) and 1.8 (◂) M. The continuous lines represent the fit of the experimental data to the model described in methods.

Figure 3 shows representative heat-induced unfolding profiles of Mth10b performed in basic running buffer containing different concentrations of GdnHCl (from 1.0 to 1.8 M). Similar with denaturant-induced unfolding profiles, as shown in the figure, increasing the temperature in the presence of GdnHCl results in unfolding of the protein by an apparent two state mechanism. Moreover, the thermal transition curves also displayed good dependence on protein concentration (data not shown).

**Figure 4:**
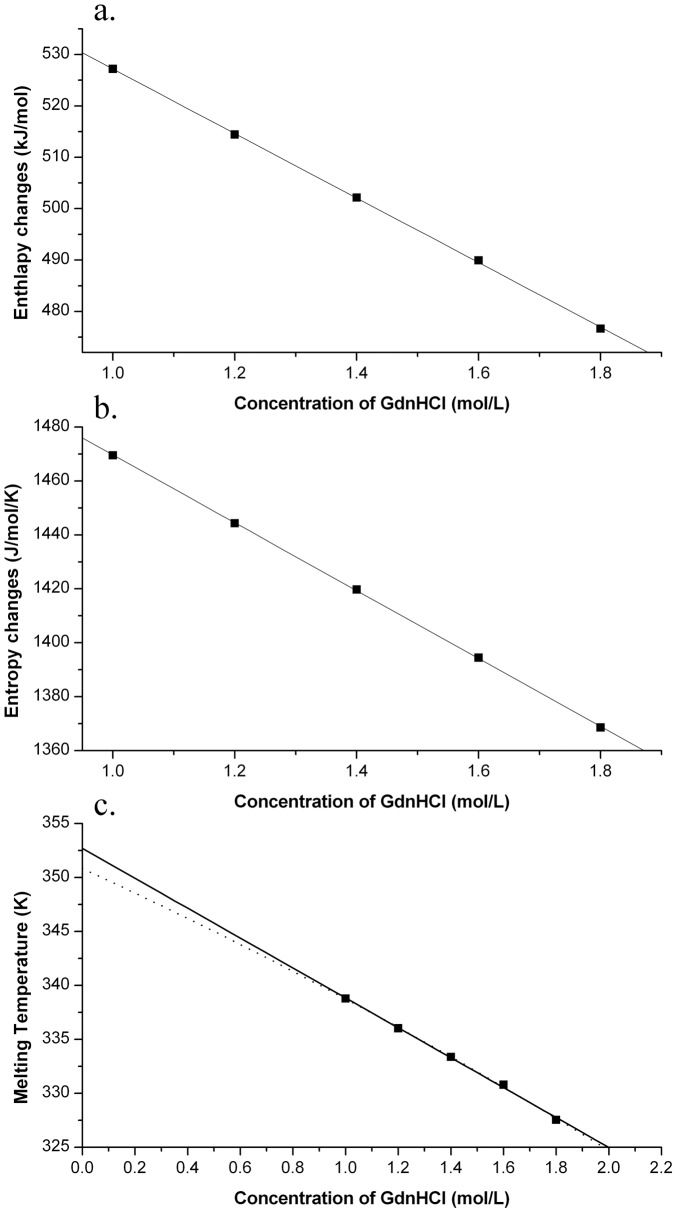
The GdnHCl dependence of the thermodynamic parameters obtained from thermal unfolding. a. enthalpy change; b. entropy change; c. melting temperature.

The heat-induced unfolding profiles were analyzed by using the two-state denaturation model described in methods: the parameters Δ*H*_m_ and Δ*S*_m_ were obtained by fitting *K*_obs_ to the van't Hoff plot. The obtained values of Δ*H*_m_ and Δ*S*_m_ appears decreased linearly with increasing GdnHCl concentration (Figure 4a and 4b), as expected (Equations 4 and 5). Linear extrapolation to GdnHCl concentration of zero gave the values of Δ*H*_m_(H_2_O) = 590.0±0.7 kJ·mol^−1^, 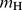 = 62.8±0.5 kJ·mol^−2^, Δ*S*_m_(H_2_O) = 1595.9±0.9 J·mol^−1^·K^−1^ and 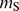 = 125.8±0.6 J·K^−1^·mol^−2^. The melting temperatures (*T*_m_) of Mth10b at different GdnHCl concentrations were calculated according to Equation 12. The obtained values of *T*_m_ also seems decreased linearly with increasing GdnHCl concentration (Figure 4c), resulting an extrapolated value of 352.7 K (79.5°C) in the absence of denaturant. According to Equation 13, the dependence of the *T*_m_ values on denaturant concentration is shown as a dotted curve in Figure 4c, which also fitted well with those obtained *T*_m_ values. Using the obtained values of Δ*H*_m_(H_2_O), 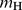, Δ*S*_m_(H_2_O) and 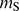, the *T*_m_ value in the absence of GdnHCl was calculated according to Equation 13. The obtained *T*_m_(H_2_O) value is 350.8 K (77.6°C), which is just slightly lower than that obtained by linear extrapolation.

### Salts-dependent urea-induced unfolding

**Figure 5:**
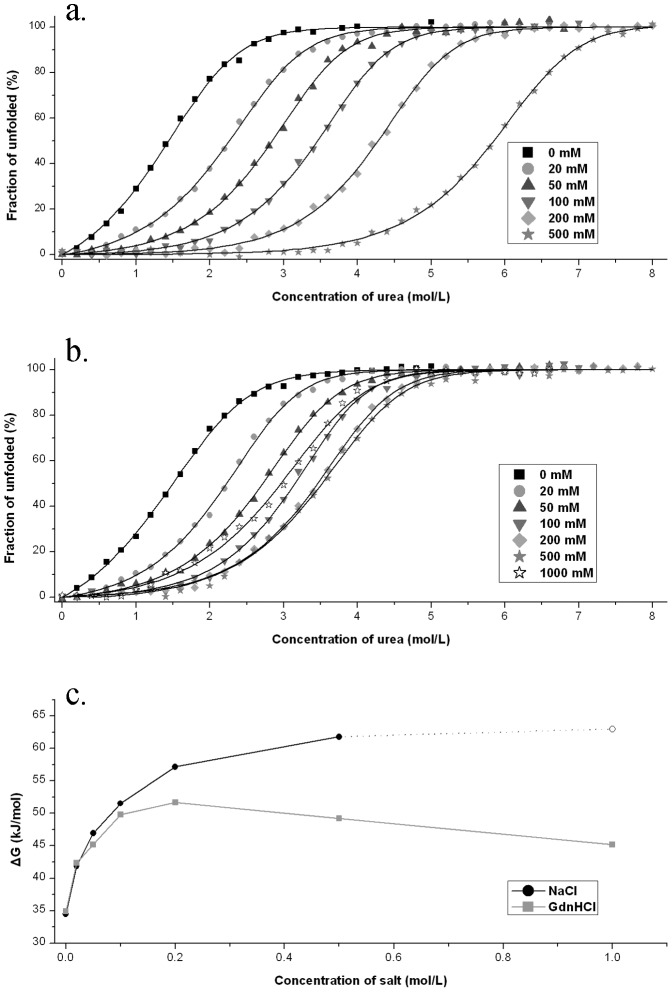
Results of salt-dependent urea-induced unfolding performed in basic running buffer at 25°C. a. Urea-induced unfolding of 15 µM Mth10b performed in basic running buffer in the presence of 0 mM (▪), 20 mM (•), 50 mM (▴), 0.1 M (▾), 0.2 M (⧫) and 0.5 M (★) NaCl. The continuous lines represent the fit of the experimental data to the model described in methods. b. Urea-induced unfolding of 15 µM Mth10b performed in basic running buffer in the presence of 0 mM (▪), 20 mM (•), 50 mM (▴), 0.1 M (▾), 0.2 M (⧫), 0.5 M (★) and 1.0 M (☆) GdnHCl. The continuous lines represent the fit of the experimental data to the model described in methods. c. The salt dependence of the free energy change (Δ*G*(H_2_O)) upon the unfolding of Mth10b obatained from urea-induced unfolding in the presence of NaCl (•) and GdnHCl (▪). The Δ*G*(H_2_O) at 1 M NaCl was substituted with the value obtained through GdnHCl-induced unfolding.

Figure 5a shows the representative NaCl concentration dependent urea-induced unfolding profiles of Mth10b performed in basic running buffer at 25°C. As shown in the figure, the resistance of the protein to denaturant urea enhanced with the increasing concentration of the additional NaCl. The transition profiles were fitted to the two-state model, as described in methods. As shown in Figure 5c, the obtained Δ*G*(H_2_O) values increased but the uptrend decreased greatly with the increasing concentration of the NaCl. In the absence of NaCl, the Δ*G*(H_2_O) value is 34.5 kJ/mol; in the presence of 0.1 and 0.2 M NaCl, the Δ*G*(H_2_O) value increased to 51.5 and 57.2 kJ/mol, respectively; in the presence of 0.5 M NaCl, the Δ*G*(H_2_O) value increased to 61.8 kJ/mol, which is almost as high as that obtained from GdnHCl-induced unfolding (63.0 kJ/mol).

Figure 5b shows the representative GdnHCl concentration dependent urea-induced unfolding profiles of Mth10b performed in basic running buffer at 25°C. As shown in the figure, at low concentrations (≤0.2 M) of GdnHCl, the resistance of the protein to denaturant urea increased with the increasing concentration of the additional GdnHCl; while at high concentrations (≥0.2 M) of GdnHCl, the resistance of the protein to denaturant urea decreased with the increasing concentration of the additional GdnHCl. The Δ*G*(H_2_O) values obtained from linear extrapolation at different concentrations of GdnHCl are also presented in Figure 4c. As shown in the figure, at low concentrations (≤0.1 M) of GdnHCl, the obtained Δ*G*(H_2_O) values increased greatly with the increasing concentration of the GdnHCl, agreeing well with the results of NaCl-dependent urea-induced unfolding; while at high concentrations (≥0.2 M) of GdnHCl, the obtained Δ*G*(H_2_O) values decreased with the increasing concentration of the GdnHCl.

## Discussion

Proteins from thermophilic and hyperthermophilic organisms are usually intrinsically thermostable. Though Mth10b is a protein from *M. thermoautotrophicum ΔH*, which is a thermophile with optimal growth temperature of 65°C, interestingly, Mth10b seems not intrinsically thermostable [36], suggesting that there are some intracellular extrinsic factors can help Mth10b remain stable under its physiological conditions. In this work, we set out to clarify the molecular mechanism of Mth10b remaining stable *in vivo* through equilibrium unfolding studies in detail.

GdnHCl and urea denaturation curves are generally employed to obtain an estimation of the conformational stability of proteins. And for most small proteins, as a denaturant, GdnHCl is found to be approximately 2.3 times as effective as urea [43]. However, results of denaturant-induced unfolding revealed that, unlike most proteins, the resistance of Mth10b to urea is much lower than to GdnHCl. The Δ*G*(H_2_O) obtained through urea-induced unfolding is just about a half of that obtained through GdnHCl-induced unfolding. Accordingly, thermal unfolding results showed that Mth10b can not remain stable at temperatures higher than 50°C in basic running buffer due to irreversible aggregation. But through heat-induced unfolding of Mth10b in the presence of GdnHCl combined with linear extrapolation method, we concluded that the melting temperature (*T*_m_) of Mth10b in the absence of GdnHCl is close to 80°C. According to this *T*_m_ value, Mth10b should remain stable at the growth temperature range of *M. thermoautotrophicum ΔH* (40–70°C) obviously. Then, what is the molecular basis leading to the huge divergence from different equilibrium unfolding experiments mentioned above.

It is well known that analysis of solvent denaturation curves can provide an estimate of the conformational stability of a protein and that GdnHCl and urea are two agents most commonly employed as protein denaturants [44], [45]. However, due to the differences in the ionic character between urea and GdnHCl, analysis of urea and GdnHCl denaturation curves may provide different estimates of the conformational stability of a protein [46], [47]. After investigated the effects of urea and GdnCl as denaturants of a synthetic coiled-coil peptide, containing variable numbers of inter- and intrahelical electrostatic interactions, Monera *et al.* suggested that the Δ*G*(H_2_O) obtained from urea-induced unfolding experiments reveals the sum effect of all kinds of interactions, while the Δ*G*(H_2_O) obtained from GdnHCl denaturation studies just represents the sum effect of all nonionic interactions due to the masking effect of GdnHCl on electrostatic interactions [47].

**Figure 6:**
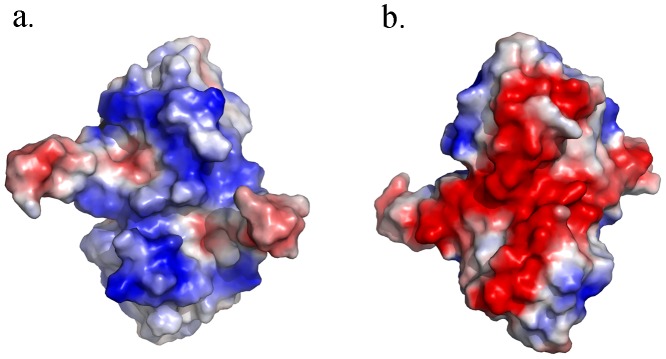
The surface electrostatic potential of Mth10b. a. the concave surface; b. the convex surface.

Recently, the crystal structure of Mth10b was solved by our laboratory (PDB code: 3TOE) [37]. Figure 6 shows the surface electrostatic potential map of Mth10b. Though Mth10b is an acidic protein, the concave surface of the molecule is surprisingly dominated by positively-charged residues (Figure 6a). Accordingly, the electrostatic potential of the convex surface is extremely negative (Figure 6b). Considering that Mth10b has an extremely uneven charge distribution, which may be involved in the intracellular molecular recognition and interaction, we conclude that the dual character of GdnHCl may be the key factor resulting in the huge divergence in the conformational stability studies of Mth10b.

To verify this hypothesis, urea-induced unfolding studies of Mth10b were further performed in the presence of different concentrations of NaCl or GdnHCl. Results of NaCl dependent urea denaturation studies showed that the salt effect of NaCl contribute to the stability of Mth10b greatly. The salt effect of NaCl enhanced with the increasing concentration of NaCl and achieved saturation at the concentration higher than 0.5 M. Accordingly, results of GdnHCl dependent urea denaturation studies showed that GdnHCl contribute to the stability of Mth10b at low concentrations as well as NaCl, but weaken it at high concentrations. This can be explained by the fact that GdnHCl is a salt as well as denaturant. At low concentrations, the salt property of GdnHCl is dominating. Gdn^+^ and Cl^−^ are presumed to shield the large numbers of electrostatic repulsions on the surface of Mth10b (Figure 6), thereby enhancing the stability of Mth10b. At high concentrations, the denaturant property of GdnHCl is dominating. Regardless of the types of electrostatic interactions present in the protein, the binding of the Gdn^+^ ions to the proteins is presumed to predominate and to push the equilibrium toward the unfolded state [45], [48]. Conversely, addition of NaCl has no obvious effect on GdnHCl-induced unfolding (data not shown).

**Figure 7:**
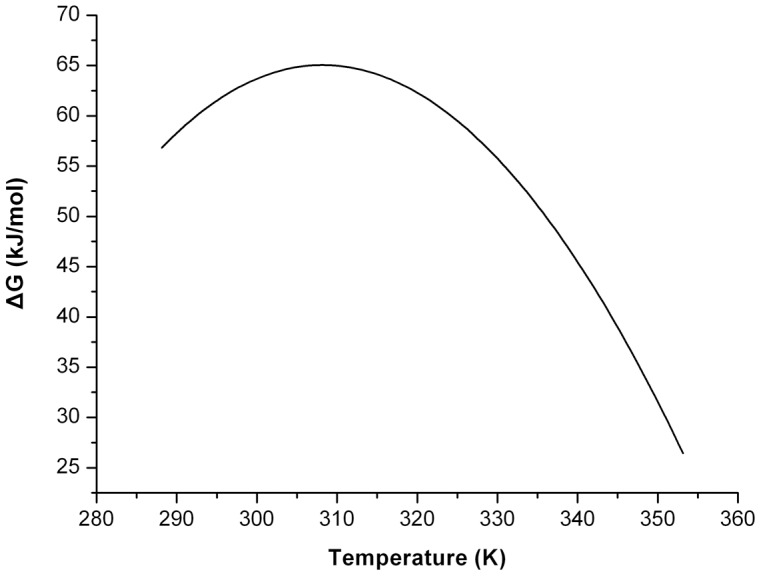
The reconstructed temperature dependent unfolding free energy of Mth10b.

These results agree well with our inference. We now return to explaining the huge divergence in the conformational stability studies of Mth10b. Due to nonionic character of urea, the Δ*G*(H_2_O) obtained from urea-induced unfolding reveals that the intrinsically conformational stability of Mth10b (34.5 kJ/mol) seems just like that of a typical mesophilic protein. For GdnHCl-induced unfolding, as the unfolding transition occurred at GdnHCl concentration higher than 1 M and GdnHCl is more effective in masking electrostatic interactions [49], the Δ*G*(H_2_O) obtained from GdnHCl-induced unfolding is the conformational stability of Mth10b under saturated salt effect (63.0 kJ/mol). Similarly, as the thermal unfolding studies of Mth10b were performed in the presence of GdnHCl no less than 1 M, the obtained parameters (Δ*H*_m_(H_2_O), Δ*S*_m_(H_2_O) and *T*_m_) through linear extrapolation are also under saturated salt effect. Therefore substituting the parameters obtained from thermal unfolding and the Δ*G*(H_2_O) at 25°C obtained from GdnHCl-induced unfolding into the Gibbs-Helmholtz equation, we got the heat capacity change (Δ*C*_P_) upon unfolding of Mth10b with a value of 68.5 J·mol^−1^·K^−1^·residue^−1^, which seems like the typical Δ*C*_P_ value of a mesophilic protein (the average Δ*C*_P_ value for mesophilis proteins is around 50 J·mol^−1^·K^−1^·residue^−1^ [50]). Then, substituting the Δ*C*_P_ and those parameters obtained from thermal unfolding into the Gibbs-Helmholtz equation, the profile of temperature-dependent free energy change upon unfolding of Mth10b under saturated salt effect was obtained (Figure 7). According to the profile, under saturated salt effect Mth10b is very stable at room temperatures (Δ*G*(H_2_O) = 63.0 kJ·mol^−1^ at 25°C) and shows more typical conformational stability at high temperatures (Δ*G*(H_2_O) = 47.6 kJ·mol^−1^ at 65°C and Δ*G*(H_2_O) = 41.5 kJ·mol^−1^ at 70°C). Considering the fact that the intracellular salt concentration of *M. thermoautotrophicum ΔH* is high to 0.7 M [51], we conclude that salt is the key extrinsic factor for Mth10b remaining stable under its physiological conditions.

Taken together, our work demonstrated that Mth10b from thermophile is not intrinsically thermostable. As the charged residues distributed extremely uneven over the surface of Mth10b, the resulting electrostatic repulsions contribute to destabilizing the protein greatly. Due to the masking effect of soluble ions on electrostatic repulsions, salt contribute to the stability of Mth10b greatly. In other word, without salt, ‘thermophilic’ protein Mth10b is just a mesophilic one.
